# Tailoring of magnetic properties of giant magnetoresistance spin valves via insertion of ultrathin non-magnetic spacers between pinned and pinning layers

**DOI:** 10.1038/s41598-018-38269-w

**Published:** 2019-02-07

**Authors:** Si Nyeon Kim, Jun Woo Choi, Sang Ho Lim

**Affiliations:** 1 Department of Materials Science and Engineering, Korea University, Seoul, 02841 Korea; 2 Center for Spintronics Research, Korea Institute of Science and Technology, Seoul, 02792 Korea

## Abstract

The low-field sensitivity of a giant magnetoresistance (GMR) spin valve can be enhanced by tailoring the bias field of the free layer because this sensitivity and bias field are known to show a strong correlation. In this study, the free-layer bias field is reduced considerably to almost zero via the insertion of an ultrathin nonmagnetic spacer between the pinned layer and the pinning layer. The spacer promotes an increase in the density of Néel walls in the pinned layer. This increase, in turn, induces domain-wall-induced magnetostatic interactions of the free poles formed on the Néel walls inside the free and pinned layers. The magnetostatic interactions result in the formation of flux closures that act as pinning sites during the magnetization reversal process and stabilize the antiparallel magnetization state between the free layer and the pinned layer by suppressing the switching of the free layer from the antiparallel state to the parallel state. Furthermore, the spacer offers an additional advantage of increasing the GMR ratio by inducing a specular scattering effect at its top and bottom interfaces. A highly improved low-field sensitivity of 12.01 mV/mA·Oe is achieved in the sample with a Cu/Pt dual spacer.

## Introduction

Giant magnetoresistance (GMR) spin valves (SVs) are extensively used in magnetic sensors such as biosensors^1^, hard-disk read heads^2,3^, and detectors of oscillations in microelectromechanical systems^4^ because of their beneficial properties of a high signal-to-noise ratio and thermal stability. These SVs are multilayered structures comprised of two ferromagnetic (FM) layers separated by a non-magnetic (NM) spacer. One FM layer has soft magnetic properties and switches freely under an applied external magnetic field. In contrast, the other FM layer has hard magnetic properties and is strongly pinned via an interface effect known as “exchange bias (*H*_ex_),” which refers to a shift in the magnetization curve away from the zero-field axis that is induced by an exchange interaction between the FM layer and an antiferromagnetic (AFM) layer at their interface^5,6^. The two FM layers spontaneously align in parallel (P) or antiparallel (AP) direction to each other depending on the applied field, and the relative P or AP alignment of their magnetizations results in a low or high electrical resistance, respectively^7,8^. It has recently been reported that a modification of the Néel wall density through variation of the pinned-layer thickness results in domain-wall (DW)-induced magnetostatic interactions, which can reduce the free-layer bias field (*H*_bias_) to an almost zero value^9^. The almost zero value of *H*_bias_ corresponds to a considerably enhanced low-field sensitivity (*S*_0_), which is of great significance for sensor applications. It has also been reported that the insertion of an NM spacer between the pinned layer and the pinning layer causes an increase in the domain nucleation density, which can affect the DW density, during the magnetization reversal process^10^. Furthermore, the single-domain-like state of the pinned layer resulting from its strongly fixed exchange-bias field^11^ possibly changes to a multidomain state upon insertion of the NM spacer between the pinned layer and the pinning layer. In the present study, two kinds of NM spacers—an ultrathin single spacer and an ultrathin dual spacer—are inserted between the pinned layer and the pinning layer with the aim of investigating the effects of an NM spacer on the magnetic properties of the free and pinned layers in GMR SVs.

## Results

### Effects of inserting non-magnetic spacers on magnetic properties

**Figure 1:**
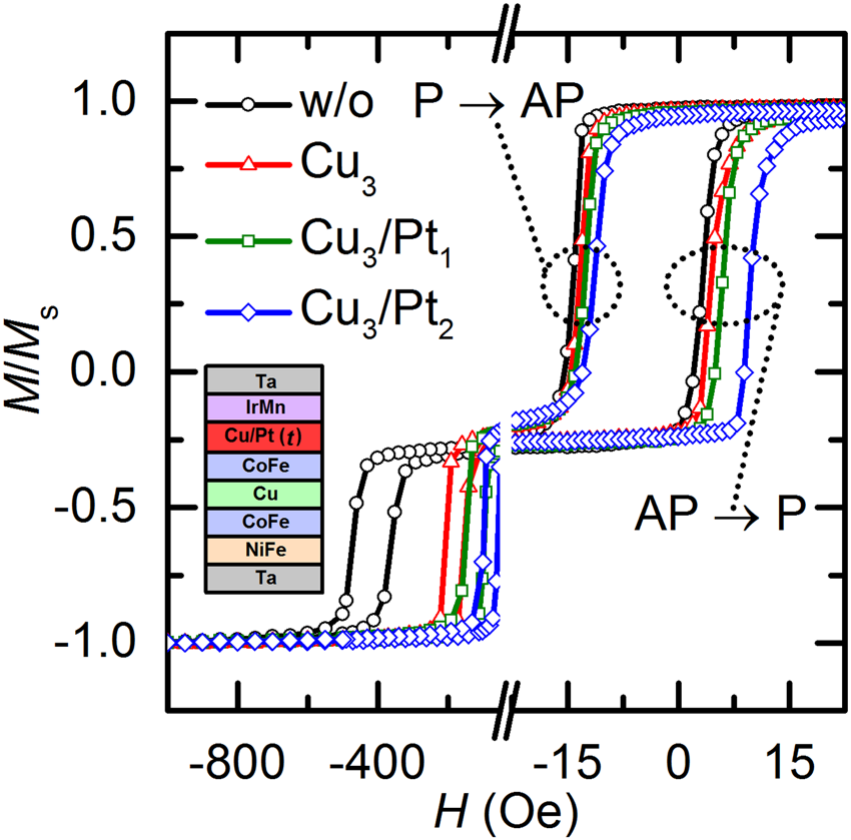
In-plane hysteresis loops of GMR SVs with variation of *t*_total_ in steps of 0.1 nm along in-plane easy direction. The results depicted are for the sample without a spacer, sample I with a Cu_3_ single spacer, and sample III with the Cu_3_/Pt_*t*-3_ dual-spacer configurations. *t* denotes the thickness of each element of the spacer and the total thickness of the spacer (=*t*_total_). The dotted circle and dotted oval indicate the free-layer switching field from the P state to the AP state and from the AP state to the P state, respectively. A schematic for the sample structure is shown in the inset.

Figure 1 shows the *M–H* curves (where *M* and *H* denote the magnetization and applied magnetic field, respectively), normalized with respect to the saturation magnetization *M*_s_, measured along the in-plane easy direction for samples I and III and for a sample without a spacer. That is, the figure depicts results for samples with different *t*_total_, i.e., for sample I with a Cu_3_ single spacer (denoted as Cu_3_ in the figure) and for sample III with the Cu_3_/Pt_1_ and Cu_3_/Pt_2_ dual-spacer configurations (denoted as Cu_3_/Pt_1_ and Cu_3_/Pt_2_, respectively, in the figure). Here, the subscript numerals represent the thickness expressed in angstroms. A schematic for the sample structure is shown in the inset. The results for the sample without a spacer are represented by the curve with black circles (denoted as “w/o” in the figure). The left and right loops indicate the magnetization reversal process of the pinned layer and free layer, respectively, where the latter shows a distinctively separate switching process resulting from a sufficient magnitude of *H*_ex_. The *H* field with a strength of 2 kOe is applied along the positive direction during post-annealing as well as during deposition, such that the loops of the pinned-layer are shifted in the negative direction. The post-annealing was performed in a vacuum at 250 °C for 10 min. The loops of the free layer are also shifted in the negative direction, and it is well known that the interlayer exchange coupling between the free layer and the pinned layer is P coupling owing to the dominant effect of Néel orange-peel coupling in GMR SVs^12,13^. The *H*_ex_ value of samples I and III is found to decrease with increasing *t*_total_. Because *H*_ex_ is a result of the interfacial nature of the pinned layer and pinning layer, its value decreases as the exchange-bias coupling weakens^14,15^, where the latter is proportional to the *t*_total_ value in this case. This weakening of the exchange-bias coupling also results in a decrease of the coercive field (*H*_c_)^16^, as is visible in the pinned-layer loops in Fig. 1. It is important to note that almost all the deposited layers have identical thicknesses between samples I and III under constant conditions; the only difference is the thickness of the inserted NM spacer layer. Nevertheless, the magnetic property of the free layer exhibits an unusual change during the magnetization reversal process, i.e., distributed values of the switching field, as indicated by the dotted circle and dotted oval in Fig. 1. Both the switching field from the P state to the AP state and that from the AP state to the P state have distributed values; however, the distribution of the latter may be wide depending on the kind and thickness of the NM spacer. The change in the values of the two types of switching fields is because the bias field of the free layer (*H*_bias_) is determined to be their respective mean. It is worth noting that the samples are fabricated at the macroscopic scale and have a square lateral geometry with a length of 10 mm. Therefore, the magnetostatic interactions of the free poles formed at the edges are negligible. However, *H*_bias_ hardly varies with the oscillatory Ruderman–Kittel–Kasuya–Yosida (RKKY)-type exchange coupling or Néel orange-peel coupling in this case. This is because the free, pinned, and sandwiched NM spacer layers are deposited with an identical thickness under constant conditions. The effect of insertion of a NM spacer between the pinned layer and the pinning layer on *H*_bias_ can possibly be explained by the DW-induced magnetostatic interactions of the free poles formed on Néel walls inside the free and pinned layers^17–19^. These interactions have been previously investigated under varying thickness of the pinned layer^9^. It has been reported that the Néel walls in the free and pinned layers couple with formed flux closures between the two layers, which act as pinning sites during the magnetization reversal process^19^. The switching field of the free layer from the AP state to the P state is more dominantly affected by the flux closures than the switching field from the P state to the AP state. Because the adjacent AFM layer forcefully fixes the pinned layer along the exchange-bias direction, the pinned layer exhibits single-domain-like behavior^11^. The free layer, on the other hand, is not pinned by such a strong exchange-bias effect, and therefore, it exhibits multidomain behavior than single-domain behavior, which results in a high density of Néel walls. The domain behavior of the pinned layer possibly changes to the multidomain type because of a considerably reduced *H*_ex_ value that results from the insertion of an ultrathin NM spacer between the pinned layer and the pinning layer. Furthermore, the inserted NM spacer acts as a source of free poles such as Néel walls and promotes the nucleation density of the domain in the pinned layer^10^. Thus, the strength of the magnetostatic interactions is dominated mainly by the Néel wall density in the pinned layer, which is modified by the insertion of the NM spacer, on account of its *mutual* nature. When the free layer is in the P state before switching to the AP state, the domains in both the free layer and the pinned layer are well aligned along the exchange-bias direction, and thus both layers are assumed to become saturated with a low Néel wall density. However, the AP state just before switching to the P state, which is the instant at which the pinned-layer magnetization reverses from the negative direction to the positive direction, is quite different from the P state, and it is estimated that the domain state of the pinned layer is indistinct, i.e., not well defined. Therefore, the samples with NM spacers between the pinned layer and the pinning layer show strong magnetostatic interactions and a wide distribution of the switching field value, especially of the switching field from the AP state to the P state.

### Some evidence of magnetostatic interactions between Néel walls

**Figure 2:**
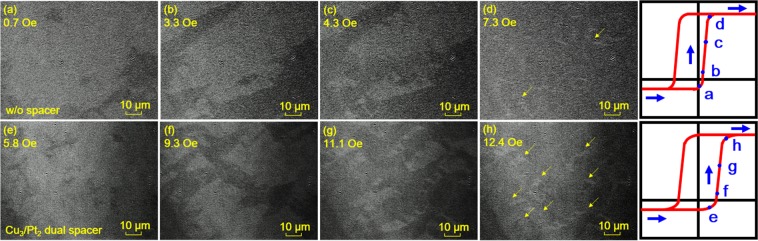
Top view of MOKE microscopy images of GMR SVs under different magnetic fields during magnetization reversal process from AP state to P state. (**a**–**d**) sample without a spacer. (**e**–**h**) sample III with Cu_3_/Pt_2_ dual spacer.

To verify the effect of the magnetostatic interactions on the free-layer switching, magneto-optical Kerr effect (MOKE) microscopy observations were performed; the results are shown in Fig. 2(a–h) for the bottom free structure consisting of NiFe and CoFe. These top-view images demonstrate the magnetic domain behavior of the free layer during the magnetization reversal process from the AP state to the P state under applied magnetic fields along the in-plane easy direction. The images were captured in the same magnetization state of each sample at four crucial magnetic field values, as indicated in the hysteresis loops of the free layer shown in the rightmost panels. The dark (bright) contrast of each image indicates a positive (negative) direction of magnetization. The results for the sample without a spacer are shown in Fig. 2(a–d), and those for sample III with the Cu_3_/Pt_2_ dual spacer are shown in Fig. 2(e–h). The free-layer magnetic moments for the former and the latter are of a similar magnitude, being 555 and 564 μemu, respectively. The difference of the magnetic moment is approximately 1% of the total magnetic moment of the entire stack of the sample. The slight increase in the magnetic moment for the sample III with the Cu_3_/Pt_2_ dual spacer is possibly ascribed to the contribution from several residual unreversed domains in the pinned layer, which will increase the Néel wall density and subsequently the magnetostatic interactions. There are two main reasons for the observation of the domain behavior of the free layer instead of that of the pinned layer. One plausible reason is a difficulty in the observation of the domain behavior of the pinned-layer on account of the small thickness of 2 nm, whereas the free layer is sufficiently thick (4.8 nm) for the observation of its domain behavior. The other reason is a clear difference in the observed free-layer domain behavior, which depends on the number of flux closures. It is noteworthy that across all the samples, almost all the different layers are deposited with the same thickness under constant conditions, the only difference being the presence or absence of the spacer and its thickness (when it is present). For the sample without a spacer, which shows *H*_bias_ of 5.3 Oe, the magnetic domains are conspicuously coarse, and the free-layer switching occurs rapidly with continuous domain wall motion, as shown in Fig. 2(b,c). In contrast, for the sample with the Cu_3_/Pt_2_ dual spacer, which shows *H*_bias_ of 0.5 Oe, the magnetic domains are very fine, as shown in Fig. 2(f,g), and they show magnetization values comparable to those in Fig. 2(b,c), respectively. Furthermore, these domains are locally reversed, during which process several residual unreversed domains remain, as indicated by the eight arrows in Fig. 2(h). This obviously demonstrates that the difference in the domain behaviors of the free layer and pinned layer is caused by the presence of the flux closures, which act as pinning sites during the free-layer switching and stabilize the AP state. As a consequence, the nucleation of reversed domains occurs randomly, and the nucleated domains are widespread because of the scattered flux-closures originating from the magnetostatic interactions. The NM spacers at the interface between the pinned layer and the pinning layer promote an increase in the Néel wall density in the pinned layer, and then, the resulting larger number of Néel walls in the pinned layer have more opportunities to couple with those present in the free layer. The results for free-layer switching from the P state to the AP state were also acquired for both the samples, though these results are not provided here. The AP-to-P switching occurs simultaneously in a narrow range of *H*, and the domain wall extends quite abruptly. This demonstrates that the magnetostatic interactions, which are promoted by the insertion of the spacer, dominantly affect the domain behavior during the switching of the free layer from the AP state to the P state. Because the pinned layer is fully saturated with a distinct domain state when it is in the P state as mentioned above, the switching of the free layer from the P state to the AP is less influenced by the magnetostatic interactions.

### Influence of inserting non-magnetic spacers on transport properties

**Figure 3:**
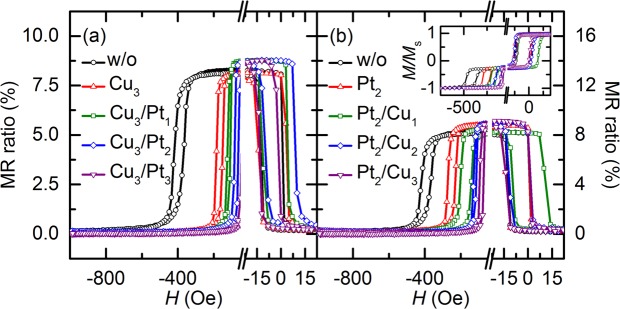
In-plane *MR*–*H* curves of GMR SVs with variation of *t*_total_ in steps of 1 Å along in-plane easy direction: (**a**) Sample without a spacer, sample I with Cu_3_ single spacer, and sample III with Cu_3_/Pt_*t*-3_ dual-spacer configurations; and (**b**) sample without a spacer, sample II with Pt_2_ single spacer, and sample IV with Pt_2_/Cu_*t*-2_ dual-spacer configurations. *t* denotes the thickness of each element of the spacer and the total thickness of the spacer (=*t*_total_). The magnetization curves for samples II and IV are shown in the inset of (**b**).

The vibrating sample magnetometer (VSM) results for *H*_ex_ and *H*_bias_ are consistent with those obtained by MOKE microscopy. This consistency is further confirmed by the transport measurements using a four-point probe method. Figure 3(a,b) show the magnetoresistance (*MR*)–*H* curves measured along the in-plane easy direction for samples I–IV. In particular, Fig. 3(a) shows the curves for sample I with the Cu_3_ single spacer and for sample III with the Cu_3_/Pt_1_, Cu_3_/Pt_2_, and Cu_3_/Pt_3_ dual-spacer configurations, and Fig. 3(b) shows the curves for sample II with the Pt_2_ single spacer and for sample IV with the Pt_2_/Cu_1_, Pt_2_/Cu_2_, and Pt_2_/Cu_3_ dual-spacer configurations (where the subscript numerals represent the thickness expressed in angstroms). The figure also shows the results for the sample without a spacer, represented by the curve with black circles (denoted as “w/o” in the figure). These results are only for the switching ranges of the free and pinned layers and are consistent with the VSM measurement results, as is expected from the results for samples I and III in Fig. 1 and samples II and IV in the inset of Fig. 3(b). The insertion of the spacer between the pinned layer and the pinning layer leads to a decrease in the *H*_ex_ and *H*_c_ values of the pinned layer and also to variations in the switching field of the free layer. The former is caused by the weakening of the exchange-bias coupling between the pinned layer and the pinning layer with increasing *t*_total_^16^, and the latter is caused by the magnetostatic interactions of the free poles inside the free and pinned layers as mentioned earlier. Moreover, an improvement in the maximum GMR ratio is observed in most of the samples with spacers, as shown in Fig. 3(a,b), except for sample I with the Cu_3_ single spacer and sample IV with the Pt_2_/Cu_1_ dual spacer. It is considered that specular scattering at the interfaces between the NM spacer and its adjacent layers contributes to an enhancement of the GMR ratio^20–22^. Because the NM spacer is deposited directly above the pinned layer, its top and bottom interfaces reflect electrons back into CoFe/Cu/CoFe multilayers, which are primarily responsible for the GMR effect. This reflection results in an increase in the mean free path of the electrons, and the maximum GMR ratio consequently increases^21^. The increment of the GMR ratio varies with the type or thickness of the spacer. It is assumed that the type or thickness of the inserted spacer determines the quality of the interface between the pinned layer and the pinning layer, and consequently, the *H*_bias_ value reduces via the occurrence of strong magnetostatic interactions and the GMR ratio improves as a result of the sharp interfaces of the NM spacer. The values of the GMR ratio are much higher than those reported for similar constituent layers with almost the same thickness: the GMR ratio of the glass substrate/NiFe (20)/CoFe (20)/Cu (22)/CoFe (20)/IrMn (60)/Ta (50) configuration (where the numerals in parentheses represent the layer thickness in angstroms) has been reported to be ~6%^23^.

### Quantitative analysis on magnetic and transport properties

**Figure 4:**
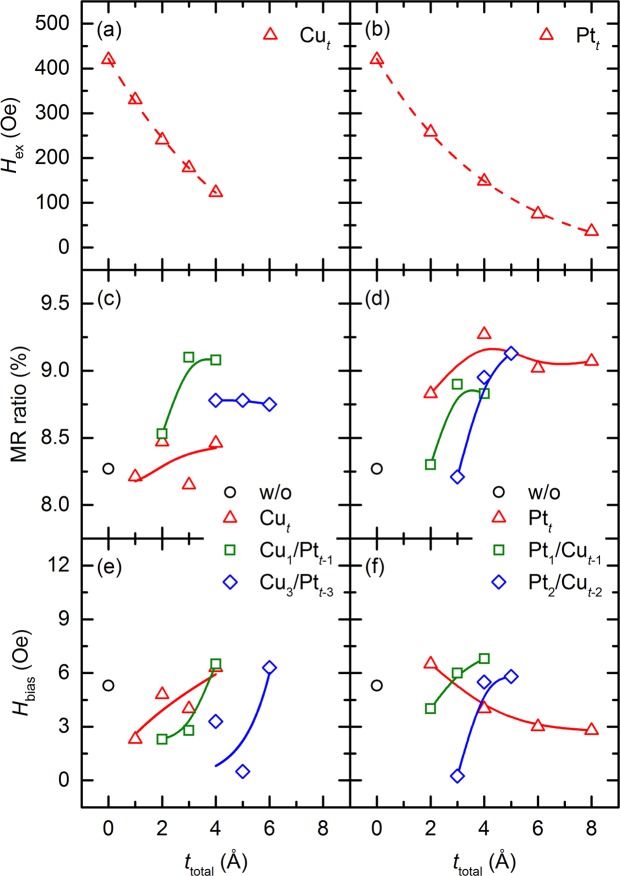
*H*_ex_, maximum GMR ratio, and *H*_bias_ as functions of *t*_total_ for four different samples: (**a**) Sample I with Cu single spacer; (**b**) sample II with Pt single spacer; (**c**,**e**) sample I with Cu single spacer and sample III with Cu/Pt dual spacers; and (**d**,**f**) sample II with Pt single spacer and sample IV with Pt/Cu dual spacers. *t* denotes the thickness of each element of the spacer and the total thickness of the spacer (=*t*_total_). The dashed red lines in (**a**,**b**) correspond to an exponential fit of measured *H*_ex_ for samples I and II, respectively.

For a more quantitative analysis of the magnetic and transport properties, some of the significant parameters, such as *H*_ex_, the maximum GMR ratio, and *H*_bias_, of samples I–IV were plotted as a function of *t*_total_, as shown in Fig. 4(a–f). The value of *H*_ex_ is defined as the horizontal shift from the origin of the hysteresis loop of the pinned layer. Here, the left panels show the results for samples I and III, in which the NM spacer adjacent to the pinned layer is Cu, whereas the right panels show the results for samples II and IV, in which the NM spacer adjacent to the pinned layer is Pt. In this figure, the subscript numerals and *t* denote the thickness of each element of the spacer and the total thickness of the spacer (=*t*_total_), respectively, in angstroms. As shown in Fig. 4(a,b), it is clear from the resultant *H*_ex_ values of sample I with a Cu single spacer and sample II with a Pt single spacer that the NM spacer has an effect on *H*_ex_ at the interface between the pinned layer and the pinning layer. Measured *H*_ex_ values exhibit a monotonous exponential decay^24^ and can be fitted with the function exp(−*t*_total_/*λ*), where *λ* denotes the decay length. The *λ* values for sample I and sample II were determined as *λ*_Cu_ = 5.4 Å and *λ*_Pt_ = 4.6 Å, respectively. These values are quite plausible because those for exchange-biased bilayers with a NM spacer have been reported to be no more than a few angstroms^15^, which are comparable to the values in the present study.

The solid lines in Fig. 4(c–f) guide the eye to the data points. As shown in Fig. 4(c,d), the GMR ratios of most of the samples, i.e., samples II and IV, increase with increasing *t*_total_, whereas those for sample I with the Cu_*t*_ single spacer (where *t* = 1–4 Å) and sample III with the Cu_3_/Pt_*t*-3_ dual spacers show an oscillation and a slight decrease, respectively, with increasing *t*_total_. In other words, insertion of the Cu layer adjacent to the CoFe pinned layer as a single spacer or a dual spacer results in a small increase in or retention of the GMR ratio. It is noteworthy that the increase in the GMR ratio, which is attributed to the specular scattering effect, is dominantly affected by the sharpness of the interface. Furthermore, the shunting effect of the spacer also influences the increase in the GMR ratio, and therefore, a nano-oxide layer with high resistivity has been conventionally used in similar GMR SVs^25^, where the high-resistivity layer prevents flow of a leakage current through the spacer. The Cu element is immiscible with the Co element^26^, possibly indicating that the Cu-Cu interactions are stronger than the ones between Cu-CoFe. This will lead to the Volmer–Weber growth^27^, i.e. the deposited Cu atoms will undergo island-type growth particularly at small *t*_total_ values. In terms of the interface morphology, insertion of the Cu spacer is likely to degrade the interface sharpness, thus leading to a weakening of the specular scattering effect at the interface. The Co/Pt interface is expected to be rough, owing to a large interpenetration of Pt into Co during the deposition^28,29^ and the miscibility between Pt and Co elements^30^. Nevertheless, high GMR values are observed from the samples with a Pt single spacer. To examine the interfacial roughness of the spacer, X-ray reflectivity (XRR) measurements are carried out for the samples having spacers of Cu_4_ and Pt_4_ (results not shown). It is found that the interface roughness is 1.22 nm for the Cu spacer and 0.487 nm for the Pt spacer. Although these values are larger than the spacer thickness (4 Å), given that the spacer thickness is extremely thin, the spacer layer might be difficult to form a fully continuous film state. Accordingly, the obtained quantitative values (1.22 and 0.487 nm) are possibly overestimated, but the relative difference between the values from Cu and Pt can qualitatively describe the interfacial quality. These results indicate that the interfacial quality is better for the Pt spacer than for the Cu spacer, thus explaining the high GMR values for the samples with the Pt spacer. The resistivity of each element is considered to mainly influence the shunting effect. The resistivities of the Cu and Pt elements are 1.68 × 10^−8^ Ω·m and 10.6 × 10^−8^ Ω·m in the bulk state, respectively. The resistivity of Pt is approximately 6.3 times that of Cu, even in the case of an ~2-nm thin film^31,32^. Thus, the GMR ratio of sample I with the Cu single spacer shows slight variations because of the rough morphology of the bottom and top interfaces of the spacer, which is considered to result from island growth and shunting through the Cu spacer—whose resistivity is lower than that of the Pt spacer—and not through the pinned-layer. In the case of the dual spacer, both the order of deposition of the Cu and Pt elements and the thickness of each of these elements play a crucial role in determining the interface morphology. It is well known that the deposition of a high-surface-free-energy metal onto a low-surface-free-energy metal causes segregation of atoms of the bottom metal layer to the surface of the top metal layer, which is accompanied by intermixing between these metals^21^. The surface-free-energies of Cu and Pt elements are 1.83 J/m^2^ and 2.48 J/m^2^, respectively^33^. Therefore, the GMR ratio of sample III with the Cu_3_/Pt_*t*-3_ dual spacers, in particular, shows a limited increase, probably because of the rough top and bottom interfaces of the spacer resulting from the segregation of Cu atoms. The GMR ratio values of sample IV with the Pt/Cu dual spacers increase with increasing thickness of the Cu spacer, because Cu atoms grow and form a smooth surface on an ultrathin Pt spacer. As mentioned earlier, the high resistivity of the Pt spacer, which is directly adjacent to the CoFe pinned layer, contributes to the larger increase in the GMR ratio of sample II with the thicker Pt spacer. Thus, the GMR ratios for sample II with a Pt single spacer are mostly high. The highest GMR ratio of 9.3% for sample II is obtained in the case of the Pt_4_ single spacer. The probable reasons for this are the sharp interfaces and the weakened shunting effect.

The *H*_bias_ results in Fig. 4(e,f) reveal that the *H*_bias_ values of samples I, III, and IV decrease as *t*_total_ decreases, though those of sample II show the complete opposite trend. It is important to note that all the layers in samples I–IV are deposited under the same conditions, and only the kind or thickness or both of the NM spacer differ in these samples. In the case of deposition of a single or dual spacer with the Cu element, the Cu atoms in an ultrathin spacer are expected to undergo island-type growth on the CoFe pinned layer because of the immiscibility of Cu with the elements of the CoFe layer as explained above. These atoms lead to an increase in the domain nucleation density at the interface between the pinned layer and the pinning layer. Thus, the increased Néel wall density in the pinned layer leads to strengthening of the magnetostatic interactions via the formation of more flux closures with the Néel walls in the free layer. Consequently, the resultant *H*_bias_ value decreases. However, deposition of a thick spacer can cause agglomeration of dispersed island-type atoms with neighboring atoms, which restores the Néel wall density in the pinned layer to the initial level. This consequently results in a weakening of the magnetostatic interactions, and then, the *H*_bias_ value becomes comparable to that of the sample without a spacer. An opposite trend is observed only for sample IV with a Pt single spacer. Because Pt atoms are miscible with the elements of the CoFe layer and these atoms penetrate to a considerable depth into the pinned layer, they act as the source of free poles, which results in an increase in the density of Néel walls that are weak when the spacer is thin. When *t*_total_ is larger than almost one monolayer, some atoms, other than those that penetrated into the pinned layer, start to accumulate at the interface between the pinned layer and the pinning layer. These atoms act as the source of free poles and hence, strengthen the magnetostatic interactions. Thus, the *H*_bias_ value of sample IV tends to decrease with increasing *t*_total_, in contrast to the trend of the *H*_bias_ values of the other samples. As a consequence, very low *H*_bias_ values are obtained for sample III with the Cu_3_/Pt_2_ dual spacer (0.5 Oe) and for sample IV with the Pt_2_/Cu_1_ dual spacer (0.25 Oe), and these values can be compared with a value of 5.3 Oe observed for the sample without a spacer.

## Discussion

**Figure 5:**
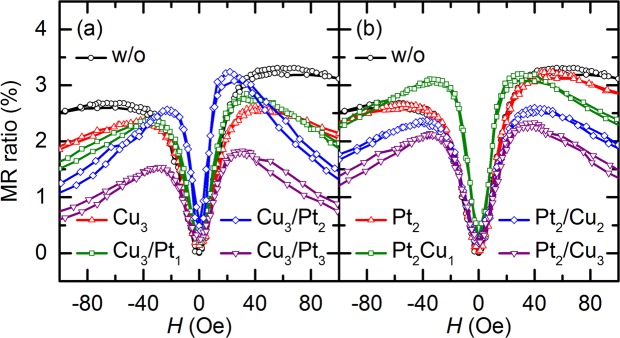
In-plane *MR*–*H* curves of GMR SVs with variation of *t*_total_ in steps of 1 Å along in-plane hard direction: (**a**) Sample without a spacer; sample I with Cu_3_ single spacer; and sample III with Cu_3_/Pt_*t*-3_ dual-spacer configurations; and (**b**) sample without a spacer; sample II with Pt_2_ single spacer; and sample IV with Pt_2_/Cu_*t*-2_ dual-spacer configurations. *t* denotes the thickness of each element of the spacer and the total thickness of the spacer (=*t*_total_).

Figure 5(a,b) show the *MR*–*H* curves measured for samples I–IV along the in-plane hard direction. Specifically, Fig. 5(a) shows the curves for sample I with the Cu_3_ single spacer and for sample III with the Cu_3_/Pt_1_, Cu_3_/Pt_2_, and Cu_3_/Pt_3_ dual-spacer configurations, and Fig. 5(b) shows the curves for sample II with the Pt_2_ single spacer and for sample IV with the Pt_2_/Cu_1_, Pt_2_/Cu_2_, and Pt_2_/Cu_3_ dual-spacer configurations (where the subscript numerals represent thickness values expressed in angstroms). The figure also shows the results for the sample without a spacer, which are represented by the curve with black circles (denoted as “w/o” in the figure). Although the results should be perfectly symmetric with respect to *H* = 0 in an ideal system, the measured transport results are slightly asymmetric. The distribution of anisotropy of the pinned layer resulting from magnetic-field annealing or an error in the measurement direction (i.e., a deviation of the angle of the applied magnetic field from the hard axis of the free or pinned layers) is considered to possibly affect the results of an actual system. To estimate the error in the measurement direction, a macrospin simulation based on the Stoner–Wohlfarth (S–W) model was carried out^34^. A comparison of the calculated and measurement results indicates that the maximum error in the measurement direction is approximately 5°. The property of magnetoresistance along the in-plane hard direction is significantly important for magnetic sensor applications because it is directly associated with two of the most important properties of a magnetic sensor: the low-field sensitivity *S*_0_ and linearity. The value of *S*_0_, which is calculated as being equal to the slope of a linear fit in the range of 0–10 Oe, differs depending on the kind and thickness of the spacer. Given that the thickness of each layer except for the spacer is identical in all configurations of samples III and IV, the insertion of the NM spacer also affects the *S*_0_ value. Either an increase or a decrease in the *S*_0_ value is observed in samples I–IV. In particular, a large increase in the *S*_0_ value is observed for sample III with the Pt_2_Cu_1_ dual spacer and sample IV with the Cu_3_Pt_2_ dual spacer, as shown in Fig. 5(b,d), respectively. These increased *S*_0_ values are primarily a result of the considerably decreased *H*_bias_ value. This is because *S*_0_ and *H*_bias_ are closely correlated with a Pearson correlation coefficient of –0.9, as reported previously^9^.

**Figure 6:**
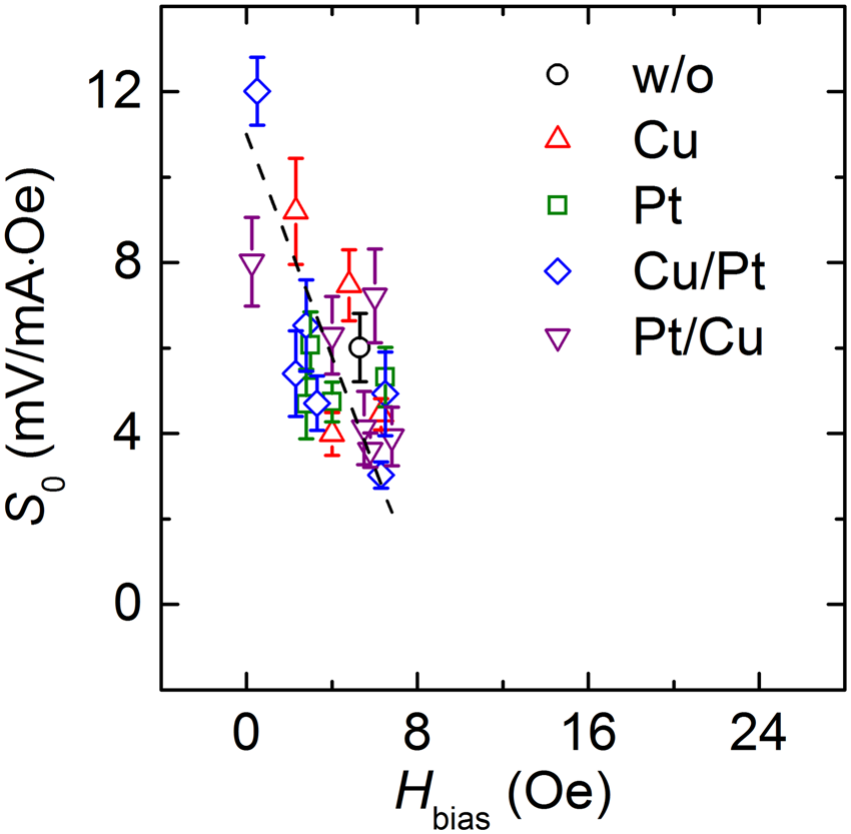
Plot of *S*_0_ as a function of *H*_bias_ for five different samples: sample without a spacer (black circles), sample I with Cu single spacer (red triangles), sample II with Pt single spacer (green squares), sample III with Cu/Pt dual spacers (blue diamonds), and sample IV with Pt/Cu dual spacers (purple inverted triangles).

For a detailed analysis of the correlation between the properties of samples I–IV, the *S*_0_ values were plotted as a function of *H*_bias_, as shown in Fig. 6. Here, the dashed black line is a guide line for an evident correlation between *S*_0_ and *H*_bias_. These two parameters show a strongly negative correlation as expected from previous results. Therefore, *S*_0_ increases with a decrease in *H*_bias_. For instance, the highest *S*_0_ value among those of samples I–IV is 12.01 mV/mA·Oe, whereas the *S*_0_ value of the sample without a spacer is 6.01 mV/mA·Oe. The magnitudes of *H*_bias_ corresponding to these *S*_0_ values are 0.5 Oe and 5.3 Oe, respectively. Meanwhile, the maximum GMR ratios for sample III with the Cu_3_/Pt_1_, Cu_3_/Pt_2_, and Cu_3_/Pt_3_ dual-spacer configurations along the hard direction show variations, as shown in Fig. 5(a), though those along the easy direction are nearly constant. It appears that not only *S*_0_ but also the maximum GMR ratio along the in-plane hard direction is affected by the magnitude of *H*_bias_. Because the free and pinned layers are mainly coupled by Néel orange-peel coupling despite a decrease in *H*_bias_ caused by the magnetostatic interactions, these layers tend to maintain the P coupling state, while the free layer or pinned layer preferentially aligns along the magnetization direction of the pinned layer or free layer, respectively. The alignment between the free layer and the pinned layer is less strongly affected by P coupling as the *H*_bias_ value approaches zero. Therefore, the free layer strongly maintains a perpendicular alignment with the pinned layer in the low-field range during its magnetization reversal process along the hard direction. For this reason, the values of the maximum GMR ratio along the hard direction and *S*_0_ increase as the magnitude of *H*_bias_ decreases. Therefore, the insertion of an NM spacer between the pinned layer and the pinning layer has significant implications for practical sensor applications because this spacer can reduce the *H*_bias_ value by manipulating the magnetostatic interactions and enhance the GMR ratio by inducing the specular scattering effect, which consequently leads to an improvement in *S*_0_.

In this study, a very high *S*_0_ value of 12.01 mV/mA·Oe was achieved for sample IV with the Cu_3_Pt_2_ dual spacer, which is twice that of the sample without a spacer. This was made possible by reducing the *H*_bias_ value close to zero with the use of the DW-induced magnetostatic interactions and by increasing the GMR ratio through a specular scattering at its interfaces with adjacent layers. A similar approach was used in the past that involved inserting a nano-oxide layer^35^. However, this approach had a limited success mainly owing to the difficulty of achieving a small *H*_bias_ value. It is believed that the present GMR SVs with very high sensitivity are of great importance in practical applications.

## Methods

GMR SVs with the following stack structure were investigated in this study: Si substrate (wet-oxidized)/Ta (50)/NiFe (30)/CoFe (18)/Cu (22)/CoFe (20)/NM (*t*_total_)/IrMn (60)/Ta (50) (where the numerals in parentheses denote the layer thickness in angstroms). NiFe/CoFe bilayers are often employed as the free-layer, as they exhibit better soft magnetic properties than a CoFe single layer^8^. Here, *t*_total_ denotes the total thickness of the NM spacer layer, which was varied from 1 to 8 Å in steps of 1 Å (or 2 Å in some cases). Among the many possible variations of the stack structure, the following four stack structures were mainly considered in this study (the variations of only the spacer layer are described here, as the rest of the layers were identical in all the stack structures): Cu (1–4) (sample I); Pt (2–8) (sample II); Cu (1 or 3)/Pt (1–3) (sample III); and Pt (1 or 2)/Cu (1–3) (sample IV). It should be noted that the Cu/Pt and Pt/Cu dual spacers were inserted between the CoFe pinned layer and the IrMn pinning layer in samples III and IV, respectively. Four different alloys were included in the stacks, which were deposited using the following alloy targets (at.%): Ni_80_Fe_20_, Co_90_Fe_10_, and Ir_21_Mn_79_. The stacks were deposited using an ultrahigh-vacuum direct-current magnetron sputtering system. The base pressure of the chamber was 7 × 10^−8^ Torr, and the Ar partial pressure during the deposition was 2 × 10^−3^ Torr. The powers applied to the targets were as follows: 15 W for Ta, 10 W each for NiFe and CoFe, and 5 W each for IrMn, Cu, and Pt. The deposition rates of the elements were as follows: 0.049 nm/s for Ta, 0.035 nm/s for NiFe, 0.026 nm/s for CoFe, 0.024 nm/s for IrMn, 0.046 nm/s for Cu, and 0.036 nm/s for Pt. These deposition rates were obtained using separately prepared thick (~100 nm) control samples. To ensure accuracy of the deposition rates, the thickness of each control sample was measured ten times using a surface profiler. The layer thickness was determined from the deposition rate by controlling the deposition time. To induce anisotropy in the free and pinned layers, an in-plane magnetic field with a strength of 90 Oe was applied during the deposition process. Post-annealing was performed in a vacuum of 5 × 10^−6^ Torr at 250 °C for 10 min. To induce an exchange bias at the CoFe/IrMn interface, a magnetic field of 2 kOe was applied along the direction of the induced anisotropy during the post-annealing process and also during the subsequent furnace cooling to room temperature. *M*–*H* hysteresis loops were measured using a VSM along the in-plane easy and hard directions. *MR*–*H* curves along the two directions were measured by the four-point probe method at a constant current of 1 mA. The magnetic domains were observed using a MOKE microscope with a spatial resolution of 1 μm. The interfacial quality was characterized by the XRR (ATX-G, Rigaku) with Cu K*α* radiation.
